# Occipital Emissary Foramina in South Indian Modern Human Skulls

**DOI:** 10.5402/2013/727489

**Published:** 2013-02-20

**Authors:** Suruchi Singhal, Roopa Ravindranath

**Affiliations:** Department of Anatomy, St John's Medical College, Bangalore 560034, India

## Abstract

An occipital emissary foramen has been traditionally described as a foramen present in the squamous part of the occipital bone at the occipital protuberance transmitting a vein that connects the confluence of sinuses with the occipital vein. The present study was done on 221 South Indian adult modern human skulls of unknown sex in the Department of Anatomy, St John's Medical College, Bangalore, India. The foramen was observed in 21/221 (9.50%) skulls, 6/21 (28.57%) to the right of, 10/21 (47.61%) to the left of, and 2/21 (9.52%) on the External Occipital Crest. It was seen more often near the posterior margin of foramen magnum rather than at the External Occipital Protuberance as has been traditionally described. A new finding is that bilateral foramina were observed in 3 skulls (14.28%). The incidence was higher than seen in other Indian population. Since it is present near the foramen magnum in most cases, knowledge of the number and position of the foramen is important for suboccipital craniotomies. The extensive connections of the veins with cranial venous sinuses may lead to intracranial infections and vice versa.

## 1. Introduction

Emissary veins traverse emissary foramina of the skull and connect venous sinuses to extracranial veins. Although they are valveless and blood may flow in both directions, flow is usually away from the brain. In ordinary usage, emissary foramina are restricted to mastoid, parietal, condyloid, and the foramen of Vesalius. An occipital emissary foramen has been traditionally described as a solitary foramen occasionally present in the squamous part of the occipital bone at the occipital protuberance [1]. It transmits the occipital emissary vein that connects the confluence of sinuses with the occipital vein. The emissary vein may also receive the occipital diploic vein [2, 3]. This traditional view has now been challenged as the foramen has in the recent studies been found more often near the foramen magnum than the External Occipital Protuberance [4–6]. The present study was done to ascertain the incidence of the foramen in unsexed adult modern human skulls of South Indian origin. The position of the foramen was also determined as a comparison of the study with the traditional and existing literature was done. The findings of the study were then correlated to possible clinical manifestations that may arise due to the position and number of emissary foramina if seen on the skull. 

## 2. Materials and Method

The study was done on 221 South Indian adult modern human skulls of unknown sex in the Department of Anatomy, St John's Medical College, Bangalore. The squamous part of occipital bone was studied for the presence of occipital emissary foramina.

The anatomical landmarks used in the study were

1. External Occipital Crest (EOC);
2. External Occipital Protuberance (EOP);
3. posterior border of Foramen Magnum (FM).

**Figure 1:**
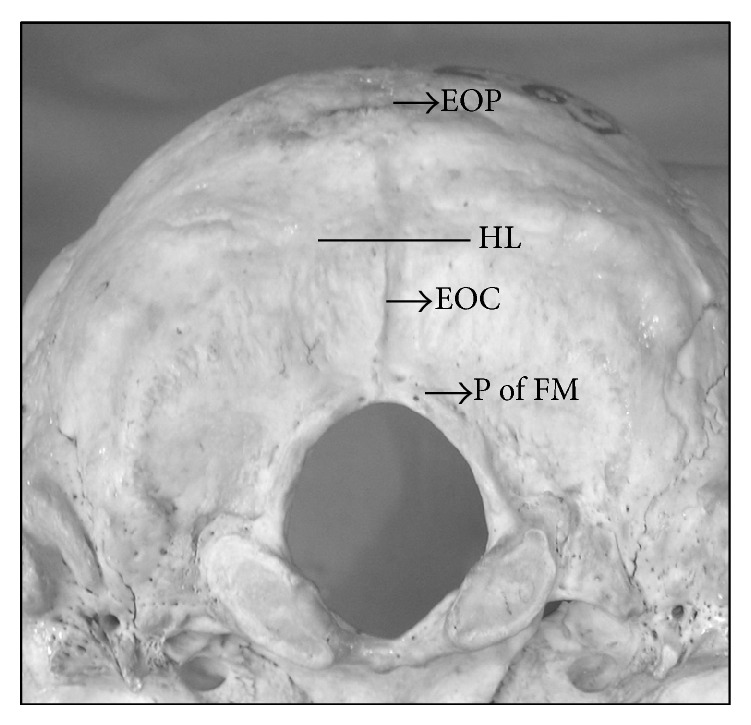
Landmarks of the study: External Occipital Crest (EOC), External Occipital Protuberance (EOP), Posterior Border of Foramen Magnum (P of FM), horizontal line (HL).

The position and number of foramina were noted. The majority of the foramina were not seen on the EOP (as traditionally described). Hence they were classified as present to the right, to the left, or on the EOC. A horizontal line was drawn midway between the EOP and the posterior margin of FM. The foramina were then classified as near the FM or near the EOP depending upon their distance from the midline (Figure 1).

A foramen was said to be present only if it transmitted a copper wire of 0.5 mm diameter and went through the bone into the skull.

## 3. Results

Out of the 221 skulls studied, the foramen was observed in 21/221 (9.50%) skulls, 6/21 (28.57%) to the right of, 10/21 (47.61%) to the left of, and 2/21 (9.52%) on the EOC. Of these two cases, one single large foramen was observed on the left side of EOC near the EOP and the second foramen on the EOC near the FM. In 3 skulls (14.28%) foramina were observed on both sides of EOC and classified as bilateral. All were near the posterior margin of FM except the one case where a single large foramen was observed on the left side of EOC near the EOP.

## 4. Discussion

Emissary foramina are a byproduct of selection of bipedalism by extant humans [9]. As an upright posture necessitated delivery of blood from the brain to the vertebral veins, venous channels like the enlarged occipital/marginal sinus system, multiple hypoglossal canals, and emissary foramina that conduct the emissary veins developed [10]. These are considered to be epigenetic adaptations for delivering blood preferentially to the vertebral plexus of veins [11]. The occipital foramen occurs at low frequencies throughout the hominid record and is generally seen near the inion [10]. 

In our study the incidence of the foramen is 9.50%. This is higher if we compare it to other studies done in Indian population (Table 1). In studies done on North Indian skulls, the incidence has varied from a single case in 214 skulls, that is, 0.46% to 2.07% [4, 8]. In Anatolian skulls the incidence is 2.6% and in Bangladeshi skulls it is 14% [5, 6]. The foramen in these studies is located near the posterior margin of FM. In a study done by Boyd, the incidence of the foramen is 1.6% and is seen near the EOP in all cases [7]. 

**Figure 2:**
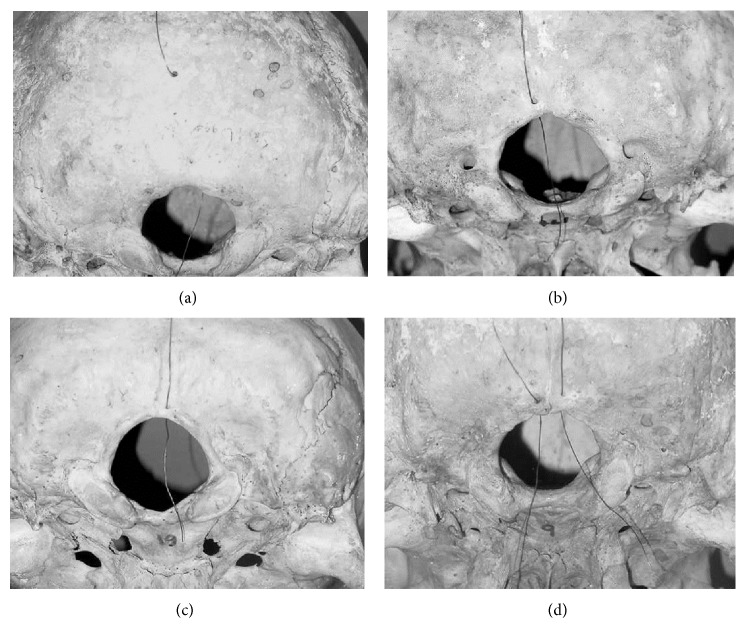
Probe in (a) occipital emissary foramen on the External Occipital Protuberance, (b) emissary occipital foramen near the foramen magnum on the left, (c) emissary occipital foramen near the foramen magnum on the External Occipital Crest, (d) bilateral emissary occipital foramina near the foramen magnum.

In our study, all but one foramen are seen near the FM. In only a single case, the foramen has been observed near the EOP (Table 2), (Figure 2(a)).

Based on the location, the occipital emissary foramen connects:

1. confluence of sinuses with the occipital vein if seen on the EOC near the EOP (1 case in the present study);
2. marginal sinuses to the occipital vein if seen on either side of FM (16 unilateral and 3 bilateral in the study) (Figure 2(b));
3. occipital sinus to occipital vein if seen on the EOC near the FM (1 case in the present study), (Figure 2(c));
4. it may also receive the occipital diploic vein.

Previous studies have reported only solitary foramina. But in our study, we have found bilateral foramina in 3 cases, all near the foramen magnum (Figure 2(d)). There is no literature available to compare with this unique finding. This implies that instead of a single vein, there may be two emissary veins connecting the occipital veins to the occipital sinus or to the marginal sinuses near the foramen magnum. These veins serve to link the vertebral venous plexus with intracranial sinuses above and vertebral, brachiocephalic, and intercostal veins below. Due to such rich venous connections, chances of spread of intracranial infections extracranially or vice versa may be accentuated. On the other hand, procedures such as radical neck dissections that involve the ligation of internal jugular veins may benefit as now intracranial venous drainage will be assured to the exterior of skull.

The position of the foramen is more important than its incidence especially in suboccipital exposure as this procedure is one of the commonest neurological practices for the management of pathologies involving the posterior cranial fossa. These procedures always include the posterior edge of foramen magnum which is either removed en bloc or piecemeal and then replaced [12]. 

In adults, the dura is tightly adherent to the skull and since it cannot be stripped easily at this location, operators have to pay particular attention to avoid tearing walls of occipital sinus. Due to the adherence of dura, during the procedure, intermittent massive bleeding from the bone or underlying dura or the sinus is expected and taken care of. But since the majority of the foramina lie near the foramen magnum (as seen in most of the recent studies and our study as against the traditional view), knowledge of their presence and position is essential to rule out unexpected bleeding that may occur when such findings are missed during initial investigations. 

## Figures and Tables

**Table 1:** Incidence of occipital emissary foramina in different populations.

S. number	Study	Solitary foramen	Bilateral foramen
(1)	Boyd (1930) [7]	24/1500 (1.6%)	Nil
(2)	Sharma et al. (1986) [8]	1/214 (0.46%)	Nil
(3)	Premsagar et al. (1990) [4]	7/338 (2.07%)	Nil
(4)	Gozil et al. (1995) [5]	8/300 (2.6%)	Nil
(5)	Hossain et al. (2001) [6]	21/150 (14%)	Nil
(6)	*Present study *	**18/221** **(9.05%)**	**3/221**

**Table 2:** Location of occipital emissary foramina in different populations.

Study (*n*)	Right of EOC	Left of EOC	On the EOC	Bilateral	Near FM	Near EOP
*Present study * (*21*)	**06**	**10**	**02**	**03**	**20**	**01**
Boyd (24) [7]	Nil	Nil	Nil	Nil	Nil	24
Sharma et al. (1) [8]	Nil	Nil	01	Nil	01	Nil
Premsagar et al. (07) [4]	Nil	Nil	Nil	Nil	07	Nil
Gozil et al. (8) [5]	Nil	Nil	Nil	Nil	08	Nil
Hossain et al. (21) [6]	10	07	04	Nil	21	Nil

FM: foramen magnum (posterior border), EOC: External Occipital Crest, EOP: External Occipital Protuberance.
